# A Region Growing Vessel Segmentation Algorithm Based on Spectrum Information

**DOI:** 10.1155/2013/743870

**Published:** 2013-11-13

**Authors:** Huiyan Jiang, Baochun He, Di Fang, Zhiyuan Ma, Benqiang Yang, Libo Zhang

**Affiliations:** ^1^Software College, Northeastern University, Shenyang, Liaoning 110819, China; ^2^Key Laboratory of Medical Image Computing of Ministry of Education, Shenyang, Liaoning 110819, China; ^3^Radiology Department, PLA General Hospital, Shenyang, Liaoning 110819, China

## Abstract

We propose a region growing vessel segmentation algorithm based on spectrum information. First, the algorithm does Fourier transform on the region of interest containing vascular structures to obtain its spectrum information, according to which its primary feature direction will be extracted. Then combined edge information with primary feature direction computes the vascular structure's center points as the seed points of region growing segmentation. At last, the improved region growing method with branch-based growth strategy is used to segment the vessels. To prove the effectiveness of our algorithm, we use the retinal and abdomen liver vascular CT images to do experiments. The results show that the proposed vessel segmentation algorithm can not only extract the high quality target vessel region, but also can effectively reduce the manual intervention.

## 1. Introduction

Nowadays, the vessel segmentation technique [1–3] is still a bottleneck of medical image processing. As vascular has an extremely complex topology structure, making most of the conventional image segmentation methods difficult to segment vascular structures accurately, so how to fast, accurately and effectively segment vascular structures from medical images becomes an important issue. However, just using simple segmentation method cannot achieve expectant purpose. And effective vascular structure enhancement is also a key point for vascular segmentation. Hessian matrix [4] is often used in enhancement filter on tubular structures. Traditional single scale-based enhancement filter is not well adapted for vascular structures with large-scale changes. Some multiscale filters such as “cole” [5] and steerable filters [6], as well as using the Hessian matrix to determine the local direction of the target [7], however, do not have good and automatic scale selection approach.

Region growing algorithm [8, 9] has small calculation complexity and high speed and is widely used in vascular image segmentation. The basic idea of the traditional growth region is to collect pixels that have similar properties together to form a region. Its performance depends largely on the position of seed points and growth conditions. On the other hand, the traditional method needs to select seed points manually in order to ensure the stability. Because the blood vessels have a very wide gray level distribution, making the traditional region growing algorithm and binary segmentation method difficult to accurately segment the vascular area, for the region growing conditions also, depends on range of the image gray level. If each seed point uses the same threshold value, the growth is easy to stop when the blood vessels become more slender.

To avoid these problems, our method is proposed and the flowchart is introduced in Section 2. Each substep of the whole method is introduced in the subsection. In Section 2.1, Hessian Matrix used for vessel enhancement is discussed. In Section 2.2, we discuss why fast Fourier transform can be used to obtain the image spectrum information and further for vascular detection specified in Section 2.3. In Section 2.3.1, we discuss how to select the seed point automatically. The most important step, the proposed vessel segmentation method, is introduced in Section 2.4. To prove the effectiveness of our method, we discuss the experiments in Section 3.

## 2. Our Method

**Figure 1:**
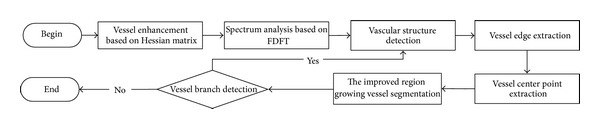
The framework of vessel segmentation.

In this paper, we present a region growing vessel segmentation based on spectrum information algorithm. The flowchart is shown in Figure 1 which will be specified in detail in this section later.

### 2.1. Vessel Enhancement Filter Based on Hessian Matrix

In this paper, we propose a vascular enhancement filter algorithms based on Hessian matrix. First Hessian matrix of image is constructed and then its feature value is calculated. Combining feature value analysis and bidirectional Gaussian kernel function, a linear multi-scale filter is created, as shown in (1) and (2) which is finally used for image enhancement.

For 2D images, the linear filter is defined as the following equation:

(1) $$\begin{array}{c} V\left(\sigma ,x\right)=\{\begin{array}{cc} 0, & \lambda_{2}>0,\\ \exp\left(-\frac{R_{B}^{2}}{2\beta^{2}}\right)\left(1-\exp\left(-\frac{\sigma^{2}}{2c^{2}}\right)\right), & \text{else}, \end{array} \end{array}$$

where the geometric ratio *R*_*B*_, defined in (2), is used to identify sheet structure. *β* is a sensitivity control parameter, here assigned to 0.5. *σ* is the scale value, and *c* depends on the gray level range, often half of the Frobenius norm of Hessian matrix. In (2), as the Hessian matrix is symmetric, we can compute three different eigenvalues *λ*_1_, *λ*_2_, and *λ*_3_, assuming that |*λ*_1_| < |*λ*_2_| < |*λ*_3_|. Consider

(2) $$\begin{array}{c} R_{B}=\frac{\text{Volume}/\left(4\pi /3\right)}{{\left(\text{Largest}\;\;\text{Cross}\;\;\text{Section}\;\;\text{Area}/\pi \right)}^{3/2}}=\frac{\left|\lambda_{1}\right|}{\sqrt{\left|\lambda_{2}\lambda_{3}\right|}}. \end{array}$$

We also construct linear filter for 3D Volume, as defined in (3):

(3) $$\begin{array}{c} V\left(\sigma ,x\right)=\{\begin{array}{cc} 0\;\lambda_{2}>0\;\text{or}\;\lambda_{3}>0, & \\ \left(1-\exp\left(-\frac{R_{A}^{2}}{2\alpha^{2}}\right)\right)\exp\left(-\frac{R_{B}^{2}}{2\beta^{2}}\right) & \\ \;\times \left(1-\exp\left(-\frac{\sigma^{2}}{2c^{2}}\right)\right)\text{else}, & \end{array} \end{array}$$

where *α* and *β* are sensitivity control parameters, here assigned to 0.5, The second geometric ratio *R*_*A*_, as defined in (4), is used for distinguishing globular structure and tubular structure. Only when the ratio value is zero or *λ*_2_ is close to zero, this means that the structure to which the pixel belongs is a globular structure instead of a tubular structure. And in general it belongs to the tubular structure when the ratio is close to 1. Consider

(4) $$\begin{array}{c} R_{A}=\frac{\left(\text{Largest}\;\;\text{Cross}\;\;\text{Section}\;\;\text{Area}\right)/\pi }{{\left(\text{Largest}\;\;\text{Axis}\;\;\text{Semi-length}\right)}^{2}}=\frac{\left|\lambda_{2}\right|}{\left|\lambda_{3}\right|}. \end{array}$$

**Figure 2:**
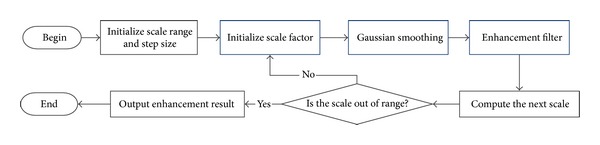
The flowchart of multiscale vessel enhancement based on Hessian matrix.

Each pixel is enhanced in a given scale range. And for each scale, all the pixels will be calculated using enhancement filter with a given scale. After traversing all the pixels, the scale will be increased by a fixed step. The flowchart of the algorithm is shown in Figure 2.

The main point of the algorithm is the convolution bidirectional Gaussian kernel function with the image, not only eliminating noises but also providing different scales for the enhancement process. Since our algorithm is based on different scale factor, many enhancement results will be output. For different outputs with different scales, we select the maximum one as the optimum enhancement result. In this paper, we use four different scales valued as 1, 3, 5, and 7.

### 2.2. Image Spectrum Analysis Based on FDFT

Spectrum analysis [10, 11] is to make Fourier transform on signal. As the general length of vascular structure is finite, we use the discrete Fourier transform (DFT) as defined in (5):

(5) $$\begin{array}{c} F\left(u,v\right)=\frac{1}{MN}\sum_{x=0}^{M-1}\sum_{y=0}^{N-1}f\left(x,y\right)e^{-2\pi (ux/M+vy/N)}. \end{array}$$

**Figure 3:**
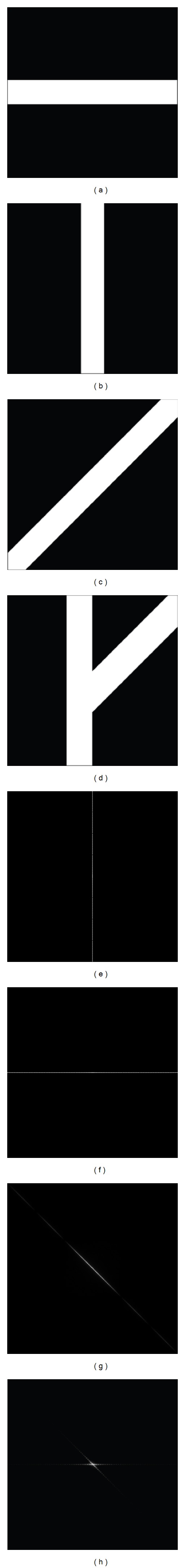
Different directional tubular structures and their spectra.

The basic idea of tubular structure detection method based on spectrum analysis is that if the image contains a tubular in its Fourier transformation spectrum images, there will be a high gray line perpendicular to the tubular structure direction. As shown in Figures 3(a)–3(c) there are three different directional tubular images; Figures 3(e)–3(g) are corresponding spectrum images after fast discrete Fourier Transform. It proves the feasibility of this idea. On another hand, just using the region growing method the vessel will have too many bifurcations or error bifurcations because of image noises. And Hessian matrix-based approach cannot easily detect the direction of tubular structure branches. But the spectrum analysis method can easily solve these problems.  Figure 3(d) is a tubular branch image and Figure 3(h) is its spectrum image, from which we can find that there still exist two high gray lines perpendicular to the branch structure. So the spectrum analysis can also be used for detecting branches. 

### 2.3. Fully Automatic Seed Points Selection Based on Spectrum Information

In this paper, we propose automatic seed selection method based on spectrum information that can ensure the stability of region growing, while significantly reducing manual intervention.

#### 2.3.1. Vascular Structure Detection Based on Spectrum Information

To extract the spectrum primary feature line and direction, first we should analyses the spectrum's feature energy which is defined as the following equation:

(6) $$\begin{array}{c} G_{\text{Energy}}\left(\theta ,\omega \right)=\int_{-\infty }^{\infty }F\left(u,v;\theta ,\omega \right)du\;dv. \end{array}$$

In (2), *G*_Energy_ (*θ*, *ω*) denotes the energy of the spectrum region which is centered on *θ* and ranges in (*θ* − *ω*, *θ* + *ω*). *F*(*μ*, *ν*, *θ*, *ω*) represents any pixel in the region. The energy is computed when the values of *θ* and *ω* is user-specified. 

For all the angle value of *θ*, the maximum of the *G*_Energy_ corresponding to the angle is the primary feature line direction. So, if there is any other peak point, you can extract the subprimary feature line and its direction. The direction is computed by (7) as follows:

(7) $$\begin{array}{c} {\vec{V}}_{\theta \leq 90}=\left(\cos\theta ,\sin\theta \right),\\ {\vec{V}}_{\theta >90}=\left(\sin\theta ,\cos\theta \right). \end{array}$$

**Figure 4:**
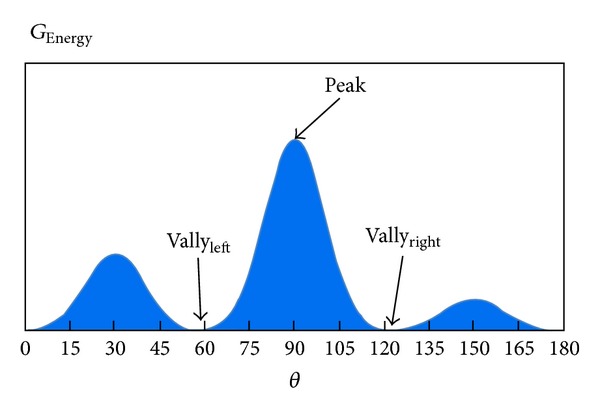
The spectral energy of vessel structure.

In a certain region of interest, if the vascular structures appear, there will be a wide range of fluctuations with the energy value of the spectrum showing a trough-peak-trough shape, as seen in Figure 4.

Here, we use (8) to detect the vascular structures in the region of interest. Consider

(8) $$\begin{array}{c} \frac{\text{Peak}-\text{Vall}{\text{y}}_{\text{left}}}{\text{Distanc}{\text{e}}_{\text{left}}}\geq \text{Threshold},\\ \frac{\text{Peak}-\text{Vall}{\text{y}}_{\text{right}}}{\text{Distanc}{\text{e}}_{\text{right}}}\geq \text{Threshold}, \end{array}$$

where peak is the peak value of *G*_Energy_, and Vally_left_ and Vally_right_ represent trough value in the left and right of the peak. Obviously Distance_left_ denotes the distance between the left trough point and the peak point. For a given threshold, if and only if (8) is satisfied simultaneously, it can be identified as vascular structures.

#### 2.3.2. Seed Points Selection Based on Edge Information

Generally, the seed points should be in the region of interest, while trying to avoid the edge of the region. To achieve this, first we use Sobel [12] operator to extract the edge information, that combined which and the obtained primary feature direction. We cut down in the direction that perpendiculars to the vascular structure's feature direction. Then we will get a similar circular cross section. After doing matched filter on it to reduce the noise and to highlight the features, we use the point that has highest gray value as the center point and also the seed point of the following region growing segmentation process.

### 2.4. The Improved Region Growing Vessel Segmentation Algorithm

#### 2.4.1. Branch-Based Region Growing

**Figure 5:**
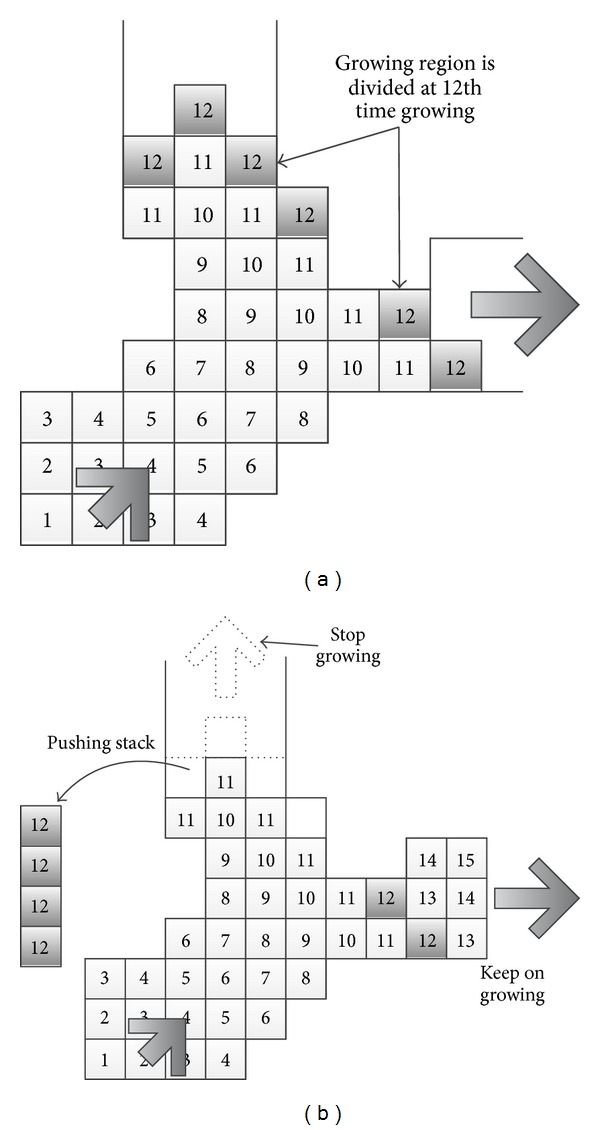
Procedure of growing at the branch connection.

Branch-based region growing algorithm takes a single branch growth strategy. That is when a branch happened during the growth, for each time only one branch grows.  Figure 5 illustrates the growing process of traditional method and branch-based method.

**Figure 6:**
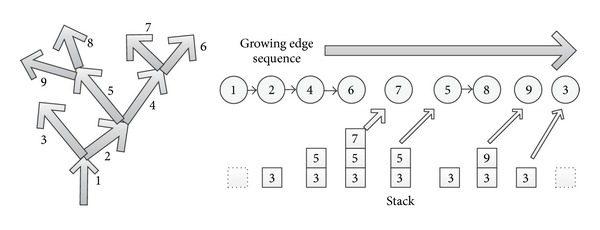
Order of the branch extraction and the contents of the stack.

Figure 5(a) shows how to grow when encountered branch for traditional method, and Figure 5(b) shows the growing rules using branch-based method. The number in the square box indicates in which growing cycle the pixel is covered. For example, the number valued 1 means the speed point. When the cycle grows from 11 to 12, we can find the pixels marked as 12 are divided into two parts, which means that there is a branch here. At this time, branch-based region growing method will only select one of the branches to continue region growing. As shown in Figure 5(b), the right branch is selected. The rest points in the left branch are pushed into the stack and will not be popped for continuing growing until the left branch comes to a complete stop. For the stack, the first point to be popped is the latest to be pushed in. This process is illustrated in Figure 6.

#### 2.4.2. Branch Detection of Vascular Structure

Adopting the branch-based strategy can change region growing conditions dynamically; that is, using different conditions for different branches can significantly improve the segmentation precisely. But there still exist some problems. For example, too many branches will reduce the speed dramatically. So it is very critical to reduce the number of branches when judging and finding branches while not affecting the accuracy of the algorithm. In each growth cycle, we apply region connectivity detection to find the branch. The following content specifies this process.

1. When a new pixel in the region starts growing, the growing target is the pixels that have the same cycle with the same period of the pixels.
2. When stopping to grow, partial pixels in the region are marked as the parent pixels for the next growing cycle.
3. Those who are not connected to each other but with the same growth cycle are identified as the branches which must be retroactive.

To reduce the excessive division of the branch and to verify the target region's connectivity, we will make the following changes.

**Figure 7:**
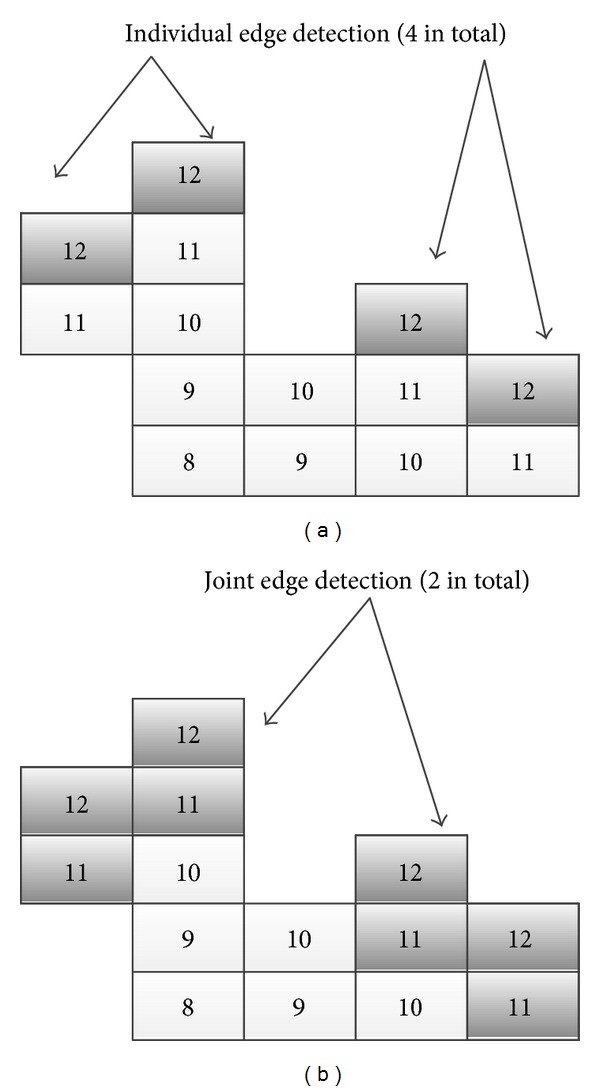
Detection of branch bifurcation: (a) only cycle 12, (b) both cycles 11 and 12.

1. We Use 8-neighborhood (2D) or 26-neighborhood (3D) (traditional region growing typically using 4 or 6-neighborhood). And the diagonal direction will also be considered for growing direction.
2. Each time growing cycle will be expanded from 1 to 2, namely, adding one time to the target growing cycle which expands the target searching region as in Figure 7.

When using four- or six-neighborhood sizes, that is, the growing range is one pixel around the seed point, this will result a lot of nonadjacent points at the end of each growth. As shown in Figure 7(a), there are four separate edges. But if using 8 or 26-neighbor sizes as growing condition, as Figure 7(b) shows, only two joint edges that consists of cycle number 11 and 12 will be detected. This effectively reduces the number of branches, but it does not affect segmentation accuracy.  Table 1 gives the relationship between the number of branches and the growing conditions.

How to dynamically change the growing conditions of the branch-based region growing algorithm, one approach is extracting the current region's gray value to update the growing condition. The computing formula is defined as follows:

(9) $$\begin{array}{c} \mu -h\sigma \leq g\left(x,y,z\right). \end{array}$$

Here *μ* is the mean gray value of the neighbor region for a period of time after each time of growing reset, *σ* represents the gray level difference, *g*(*x*, *y*, *z*) is the pixel value of coordinate (*x*, *y*, *z*), and *h* is a parameter that controls the growth. For vessel segmentation, we use (10) to compute the value of *μ*. Consider

(10) $$\begin{array}{c} \mu_{i}=\{\begin{array}{cc} c_{\min}+kd_{i} & \left(d_{i}<d_{c}\right)\\ c_{\max}+kd_{c}\; & \left(d_{i}\geq d_{c}\right). \end{array} \end{array}$$

Here *μ*_*i*_ represents the average gray level of the *i*th branch, and *d*_*i*_ is the average thickness. *c*_min_ denotes the minimum limit of the gray of the region of interest, and *c*_max_ denotes the maximum. *K* expresses exclusion rate (if equals to 0, then all branches are accepted). *d*_*c*_ expresses the upper limit of vessel thickness range and makes sure of the linear relationship between vessels' gray value and thickness.

## 3. Experiment Results and Discussions

### 3.1. Retinal Vascular Segmentation Experiment

This experiment focuses on the improved region growing method based on spectrum analysis and branch strategy without image enhancement procedure. The experiment uses two group of retinal vascular images, one simple group and another complex group. Experiment one uses complex data from STARE (Structured Analysis of the Retina) database which contains 20 medical retinal images and 10 kinds of retinal disease pathology. All of the image data are from TopCon TRV-50 camera, with the angle of 350 degrees, 700 × 605 pixels, and view field size of 650 × 550 pixels. We use manually vascular structure segmentation result by experts as the golden standard. We also use different traditional segmentation methods for segmentation and compare them with our method.

**Figure 8:**
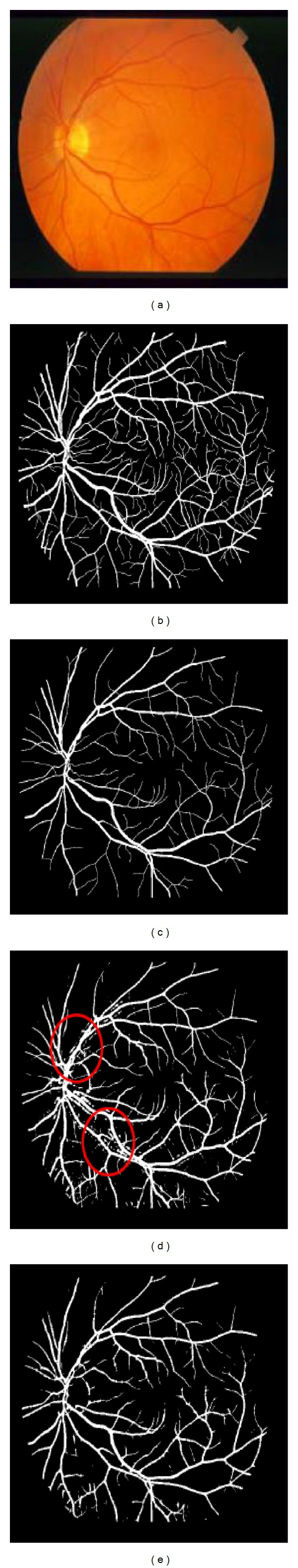
Segment results: (a) original image, (b) manual segment, (c) proposed algorithm, (d) traditional region growing algorithm, and (e) line operator edge detection algorithm.

We compare traditional region growing method and edge detection based on Sobel segmentation method with our method. The result is shown in Figure 8.

From Figure 8 we can intuitively see that the result of manual segmentation has small blood vessel branches. Comparing with it, our method is still not that accurate one but is more accurate than other methods. Traditional region growing may lead to oversegmentation in overlapping area. Seen from Figure 8(d), the red circle shows some overlap regions and scattered frags. Sobel-based edge detection method is good at segmenting clear vessels but not for small ones. We also give qualitative evaluation of those methods. The accuracy is defined as the ratio between the result and golden standard. Some accuracy is given by STARE database.  Table 2 shows the accuracy and computation time of different methods.

Table 2 shows that our method is better than the other methods in accuracy but is just faster than the traditional level set method. So it still needs to be improved in computation complexity. Comprehensively speaking, our method still has a good efficiency.

**Figure 9:**
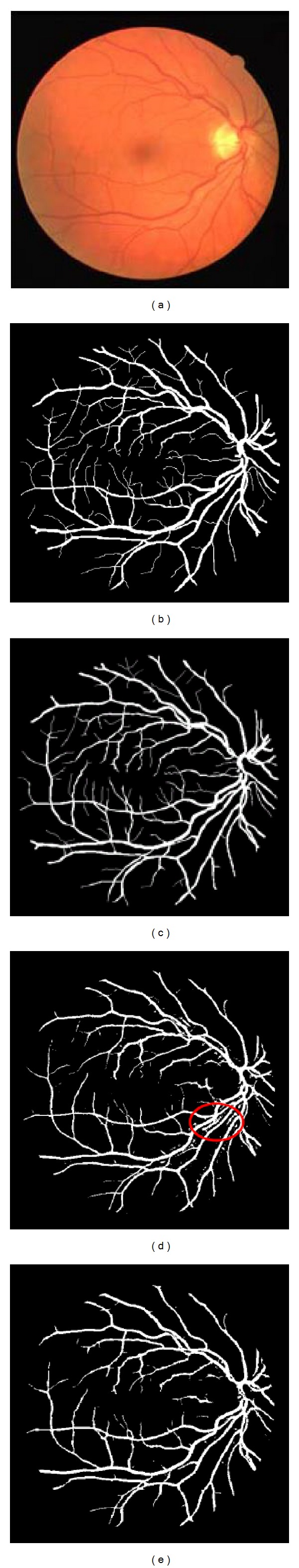
Segment results: (a) original image, (b) proposed algorithm, (c) proposed algorithm, (d) traditional region growing, and (e) line operator edge detection.

Experiment two uses a relatively simple structure of retinal vascular images from a newer database named DRIVE (Digital Retinal Images for Vessel Extraction). The results are given in Figure 9.

Figure 9 shows that our method is close to golden standard, which means that the accuracy is improved when reducing vessel branches, but at the same time it still has deficiency in segmenting vessels which are not visible to the naked eyes.  Table 3 shows the qualitative comparison results.

Table 3 shows that our method is better than other methods in accuracy, but it still needs to be improved in time efficiency. All the experiments show that our method is accurate and robust.

### 3.2. Liver Vascular Segmentation Based on Image Enhancement

The experiment data is from a large hospital's 64-slice CT scan with 0.5 mm space. The image resolution is 512 × 512 × 109. To improve processing speed, we rescale the image gray level to 256.

**Figure 10:**
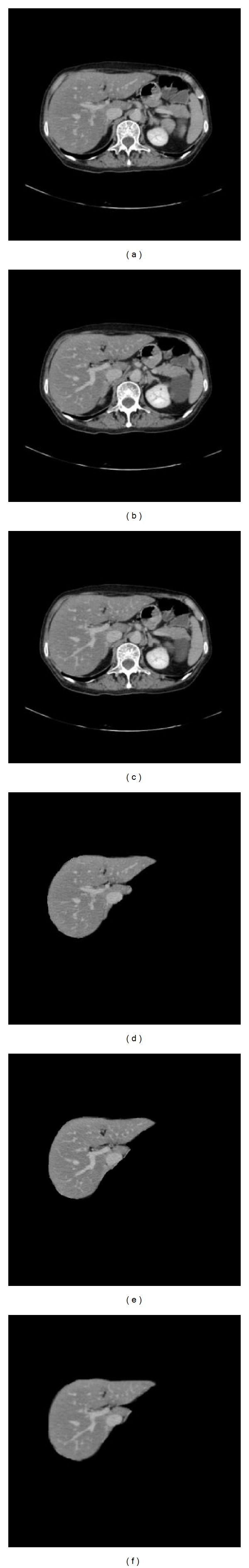
Segmentation results of liver sequences.

For liver image sequences, at first, we use the manual segmentation result as the initial contour of the latter active contour model segmentation process.  Figure 10 shows the first three original slices from Figures 10(a) to 10(c) and their segmentation results Figures 10(d)–10(f).

**Figure 11:**
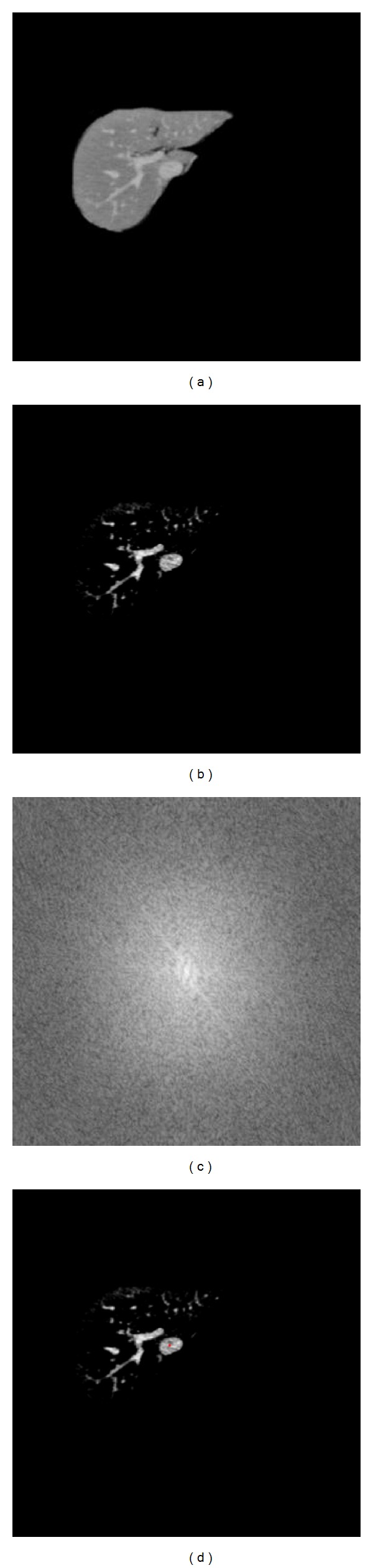
The image enhancement and seed point selection result of vessels.

Secondly, for the use of the improved region growing algorithm for liver vessel segmentation, we just select one seed point. In this way, we can select only a good segmented liver slice and then process it with Hessian matrix-based image enhancement filter. Enhanced results are given in Figure 11(b).

**Figure 12:**
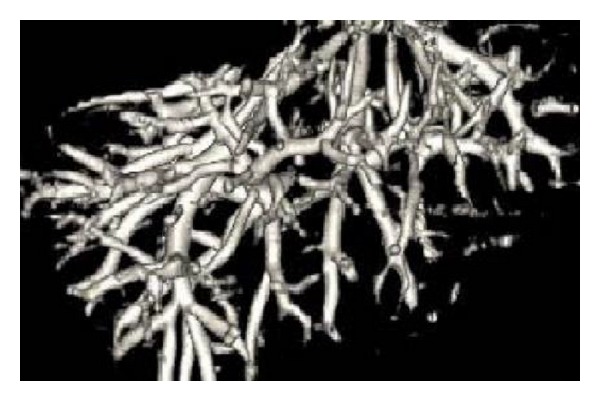
The 3D reconstruction result of vessel structure.

After image enhancement, we use the mentioned automatic seed points selection approach based on spectrum information. Spectrum image and the final selection of seed points are given in Figures 11(c) and 11(d) (red point). Finally, we give the 3D reconstruction results as shown in Figure 12.

From the reconstruction result, we can see that for 2D image vessel segmentation our approach can achieve good results, but it still has deficiency in 3D segmentation as some rupture and overlap region of vascular cannot be shown clearly. This may be due to the deficiency of region growing method and CT spaces. So it still needs to be improved in future research.

## 4. Conclusions

In this paper, spectrum information-based region growing vessel segmentation algorithm is proposed. First, wavelet transform is used for the image denoising preprocessing, and then fast Fourier transform is used to obtain the image spectrum information for analyzing, according to which vascular structure is detected and its primary feature direction and other secondary feature directions are extracted and combined with the edge information for computing its center point. Which is also the seed point of the improved region growing algorithm. At last an improved region growing algorithm is used to segment the entire vascular structures. In the experiment section we use the retinal vascular image for segmentation and compare our method with some traditional vessel segmentation methods. The experiment has proven that our method is robust and more accurate, but the efficiency needs to be improved.

## Figures and Tables

**Table 1:** Relationship between the number of branches and the growing condition.

Step	Size	No. of branches
1	6	96390
1	26	204
2	6	159
2	26	115

**Table 2:** Quality evaluation of segmentation result in experiment one.

Method	Ratio	Time (s)
Our method	0.9173	11.4
Traditional region growing	0.8511	10.1
Linear edge detection	0.8793	9.8
Traditional level set method	0.8914	14.5

**Table 3:** Quality evaluation of segmentation result in experiment two.

Method	Accuracy	Time (s)
Our method	0.9214	15.4
Traditional region growing	0.8635	14.1
Linear edge detection	0.8993	13.8
